# The Classical Pathways of Occipital Lobe Epileptic Propagation Revised in the Light of White Matter Dissection

**DOI:** 10.1155/2015/872645

**Published:** 2015-04-30

**Authors:** Francesco Latini, Mats Hjortberg, Håkan Aldskogius, Mats Ryttlefors

**Affiliations:** ^1^Department of Neuroscience, Section of Neurosurgery, Uppsala University Hospital, 75185 Uppsala, Sweden; ^2^Department of Medical Cell Biology, Uppsala University, Uppsala, Sweden; ^3^Department of Neuroscience, Regenerative Neurobiology, Uppsala University, Uppsala, Sweden

## Abstract

The clinical evidences of variable epileptic propagation in occipital lobe epilepsy (OLE) have been demonstrated by several studies. However the exact localization of the epileptic focus sometimes represents a problem because of the rapid propagation to frontal, parietal, or temporal regions. Each white matter pathway close to the supposed initial focus can lead the propagation towards a specific direction, explaining the variable semiology of these rare epilepsy syndromes. Some new insights in occipital white matter anatomy are herein described by means of white matter dissection and compared to the classical epileptic patterns, mostly based on the central position of the primary visual cortex. The dissections showed a complex white matter architecture composed by vertical and longitudinal bundles, which are closely interconnected and segregated and are able to support specific high order functions with parallel bidirectional propagation of the electric signal. The same sublobar lesions may hyperactivate different white matter bundles reemphasizing the importance of the ictal semiology as a specific clinical demonstration of the subcortical networks recruited. Merging semiology, white matter anatomy, and electrophysiology may lead us to a better understanding of these complex syndromes and tailored therapeutic options based on individual white matter connectivity.

## 1. Introduction

Firstly described in 1879 in a patient with a parieto-occipital tumour [1], occipital lobe epilepsy (OLE) is still nowadays considered a rare condition that represents less than 2–13% of extratemporal epilepsies [2–5].

Typical symptoms of OLE are visual auras and/or elementary visual hallucinations (EVHs), ictal blindness, contralateral eye and head deviation, eye movement sensations, blinking, and eyelid fluttering [6–9]. Despite this commonly accepted semiology, the diagnosis remains a problem in some cases. The seizure onset in the posterior cortex (parietal, occipital, and posterior temporal lobe) may be difficult to prove with surface electroencephalographic techniques. The epileptic propagation tends to spread rapidly to other regions in the brain up to 50% of the cases [10, 11], especially towards temporal and frontal areas [7, 12, 13], and for this reason (the semiology) is sometimes misinterpreted.

For a definitive discrimination of OLE, invasive video-EEG monitoring, using intracranial subdural and/or depth electrodes, is often necessary [9, 14–16].

Decision making for surgical treatment of drug-resistant OLE patients depends mainly on the existence of a structural lesion visible on magnetic resonance imaging (MRI). Good results are also reported with partial resection of dysplastic lesions and in some cases a favourable outcome is described in patients with a normal MRI who underwent surgical resections guided by intracranial electrodes monitoring [11, 17, 18]. The white matter connectivity seems to play a crucial role in both epileptic patterns and tailored therapeutic options.

A crucial role of the primary visual cortex has been emphasized for a long time in OLE and the relationship between the localization of the epileptic focus and the calcarine fissure seemed to determine the site and type of seizure spread [11, 13, 19]. The inferior longitudinal fasciculus (ILF) and inferior fronto-occipital fasciculus (IFOF) are still classically described as the two main white matter bundles responsible for facilitating the epileptic propagation in OLE because of their close spatial relationship with the optic radiation and the primary visual cortex (V1) [18, 20].

For this reason, the most common concern in occipital lobe surgery is aggravation of existing or creation of new visual field defects, so despite the successful results achieved with epilepsy surgery in both adults and children [11, 21–24], reports of such resections in the literature are rare (<5% of patients) [17, 25–27].

Encouraging data from tailored resection in OLE seem to have important implications for the decision making and the expected quantitative damage to the visual pathways [9, 23, 24, 28]. It seems that a more specific sublobar categorization of the resection within the occipital lobe may support a more selective disconnection of the segregated and hyperactivated bundles underlying the occipital area.

The aim of this paper is to discuss the classical pathways of occipital epileptic propagation in the light of new insight in white matter (WM) connectivity in order to better understand the complex hierarchical segregation of the occipital white matter and the different direction of epileptic propagation with its crucial surgical implications.

## 2. Methods

Ten cerebral hemispheres obtained from human cadavers donated to the Department of Medical Cell Biology, Section for Anatomy studies at Uppsala University, were enrolled in this study. All individuals donating had given written consent for use of the whole cadaver for biomedical research and education in a testimonial donation letter. The study protocol was filed with the application for ethical vetting of research involving humans to the local ethical review board in Uppsala (Dnr 2014/468).

Each brain was fixed with intra-arterial injection of 12% formalin solution within the first week after death using a perfusion device. After this procedure the brains were then carefully extracted and put in 10% formalin for at least 24 hours. The pia mater, arachnoid membrane, and vascular structures were then carefully removed under microscopic magnification and the hemispheres were frozen at −15°C–−20°C for 6–10 days and then slowly defrosted for 12 hours. Before the start of dissection, the superficial anatomy of the sulci and gyri was studied in detail. The specimens were dissected in a stepwise manner, from lateral surface to the medial structures and from mesial and basal surface to the ventricle, with a modified fiber dissection technique with respect to the technique described by Latini [29]. Microscopic metal dissectors and thin wooden spatulas were used in the initial steps of the dissection, to split or partially peel away the brain cortex, preserving the most superficial intracortical and subcortical fibres of the lateral, basal, and mesial surface of the brain. Subcortical, intralobar, associative, and projection fibres were exposed until the optic radiation (OR) on lateral and basal dissection and until the callosal-tapetal fibres on the mesial surface of the occipital lobe in each specimen. The dissections were performed under microscopic magnification (up to 4x). Between each dissection session the specimens were placed in 5% formalin.

## 3. Results

### 3.1. Dissection of the Lateral Surface

**Figure 1:**
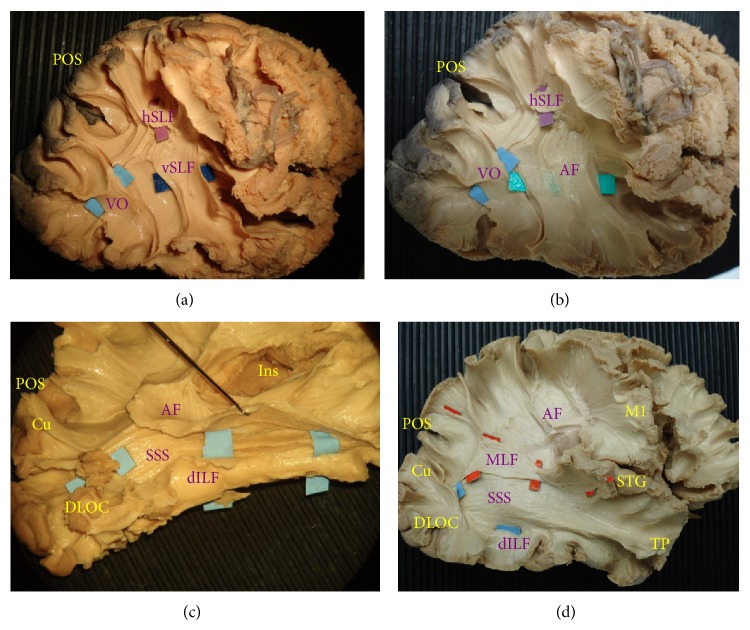
(a) Lateral view of a right hemisphere after the dissection of the superficial white matter layers of temporo-parieto-occipital region. The first bundle identified is the vertical component of the superior longitudinal fasciculus (vSLF/dark blue), which connects the supramarginal gyrus and angular gyrus to the temporo-occipital junction. Posterior to the vSLF, the vertical occipital fasciculus (VO/light blue) connects the superior occipital gyrus to the fusiform gyrus. At the level of the supramarginal gyrus the vSLF partially overlaps with the horizontal component of SLF (hSLF/purple), which runs from the parietal region to the frontal region. (b) After vSLF has been removed the arcuate fasciculus (AF/cyan) is exposed running from the frontal region through the perisylvian region with white matter terminations underneath the temporo-occipital junction and it ends at the level of the middle and inferior temporal gyrus. (c) The temporal terminations of the AF are overlapping with the temporal portion of the dorsal inferior longitudinal fasciculus (dILF/light blue). The AF has been retracted completely exposing the course of dILF. The cuneal (Cu) branch and the dorsolateral occipital cortex portion (DLOC) run in one bundle (dILF) at the level of the temporo-occipital junction lateral to the SSS and straight to the temporal pole area. (d) Medial to the AF, the middle longitudinal fasciculus (MLF/orange) is exposed at the connection between the posterior insular cortex (Ins) and the superior temporal gyrus (STG). This bundle runs from the superior temporal gyrus within the sagittal stratum of Sachs (SSS) to the parieto-occipital sulcus region (POS). At the level of the cuneal region (Cu) the dILF terminations are partially overlapping the MLF terminations.* M1*:* primary motor cortex (precentral gyrus); Ins: insula; TP: temporal pole*.

**Figure 2:**
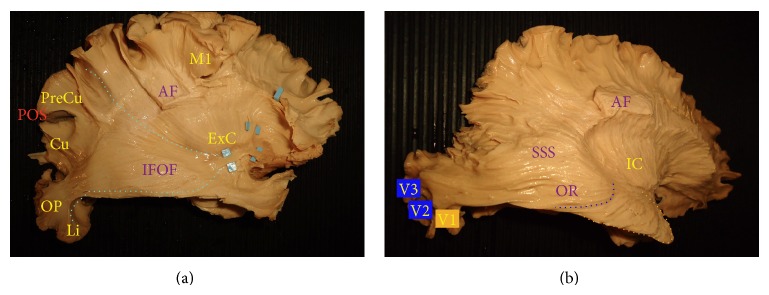
(a) Lateral view of the right hemisphere once the MLF and the insular cortex have been resected. The Inferior fronto-occipital fasciculus (IFOF) is completely exposed at the level of the external capsule (ExC). The posterior originating branches run from precuneus (PreCu), cuneus (Cu), occipital pole (OP) region, and lingula (Li) within the sagittal stratum of Sachs (SSS) towards the frontal lobe. Light blue dotted lines represent the spatial distribution of the posterior termination of the IFOF. (b) After the resection of the IFOF on the lateral surface of the brain, the optic radiation (OR) is exposed within the deep part of the SSS. According to Alvarez et al. [35] the dark blue line represents the anterior margin of the dorsal component of the OR, which will end at the level of the dorsal portion of extrastriate visual cortices (V2 and V3). The orange dotted line represents the anterior margin of the ventral component of the OR fibres that runs inferiorly and lateral with respect to the lateral ventricle to end at the level of primary visual cortex (V1).* M1: primary motor cortex (precentral gyrus); IC: internal capsule; AF: arcuate fasciculus fibres left as superficial landmark*.

The dissection of the lateral surface started with the exposure of the indirect (vertical) component of the superior longitudinal fasciculus (vSLF) (Figure 1(a)). This bundle, which connects the angular gyrus (AG) to the region of the temporo-occipital junction on the lateral surface, was considered the anterior and superficial edge to the occipital connectivity in our dissections. Medial to the vSLF the arcuate fasciculus (AF) was exposed with its c-shaped course connecting the infero-lateral temporo-occipital (T-O) region to the perisylvian region and frontal lobe (Figure 1(b)). Posterior to vSLF and AF the vertical occipital fasciculus (VO) was exposed (Figures 1(a)-1(b)). This bundle runs from the superior occipital gyrus with an oblique and caudal direction to the fusiform gyrus. Deeper and more posterior with respect to the vertical occipital fasciculus the dorsal terminations of the inferior longitudinal fasciculus (dILF) were exposed at the level of the dorsolateral occipital cortex (DLOC) and just below the parieto-occipital sulcus (POS) in the cuneal region. This portion of the ILF complex runs lateral and inferior to the sagittal stratum of Sachs (SSS) until the infero-lateral part of the temporal pole, partially overlapping at this level with arcuate fasciculus terminations [29–31] (Figure 1(c)). At the level of cuneal area (Cu) the dILF fibres cover part of the posterior terminations of the middle longitudinal fasciculus (MLF), which runs from the parieto-occipital sulcus region within the SSS and medially to the AF to the white matter underneath the superior temporal gyrus [32]. Medial to the MLF at the level of the external capsule (ExC) the inferior fronto-occipital fasciculus (IFOF) was isolated as classically described [31, 33, 34] (Figure 2(a)). Medial to the IFOF within the sagittal stratum of Sachs and caudal to the temporal portion of lateral ventricle, the optic radiation (OR) and the Meyer's loop (MeL) were exposed completely until the visual cortex posteriorly (Figure 2(b)).

### 3.2. Dissection of the Ventral Surface

**Figure 3:**
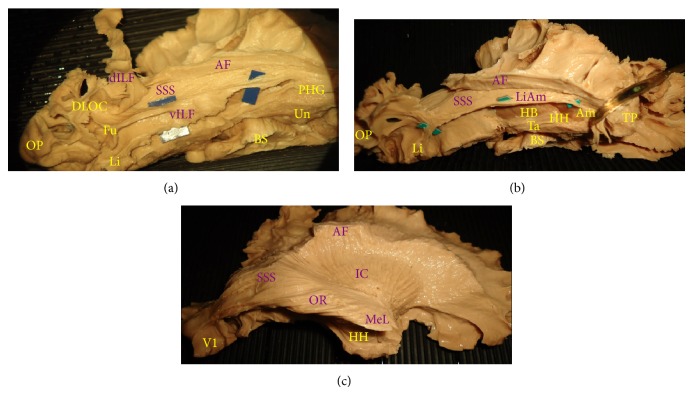
(a) Basal view of the right hemisphere showing the ventral portion of the ILF (vILF) with its double originating branches from the fusiform gyrus (Fu) and superficial lingual gyrus (Li). The bundle connects these two posterior basal regions to the parahippocampal gyrus (PHG). (b) Basal view of a right hemisphere after the removal of the dILF and vILF. The lingual-amygdala bundle (Li-Am/green) is completely exposed and its relationship with the ventricle and the sagittal stratum of Sachs (SSS) cranially is demonstrated. The Li-Am connects the lingual gyrus (Li) in an arch shaped bundle, which follows the wall of the lateral ventricle and at the level of the tip of the temporal horn turns medial to terminate in the inferolateral portion of the amygdala (Am). (c) Inferolateral view of a right hemisphere with the optic radiation (OR) completely exposed. The temporal loop of Meyer (MeL) is prominent anteriorly with respect to the other fibres. The optic radiation fibres run within the internal layer of the sagittal stratum of Sachs (SSS), inferior and lateral to the lateral ventricle, and turn medially to end at the level of the primary visual cortex (V1). The arcuate fasciculus (AF) and the internal capsule (IC) can be considered as superficial and deep landmarks, respectively.* AF: arcuate fasciculus; BS: brain stem; HB: hippocampus body; HH: hippocampus head; OP: occipital pole; Ta: tapetal fibres; TP: temporal pole; Un: uncus*.

Once the “U” fibres have been removed from the fusiform subcortical region at the level of collateral sulcus, a ventral branch of inferior longitudinal fasciculus (vILF) was isolated (Figure 3(a)). These fibres, arising from the posterior occipito-temporal gyrus, are located inferiorly and medially to the dILF, IFOF, and sagittal stratum previously described. This ventral branch runs from posterior and basal occipital region forward and slightly laterally, until the temporal pole following the course of lateral ventricle. This ventral pathway represents the infero-lateral wall of lateral ventricle for its entire course, and beyond the temporal horn it reaches the subcortical region of parahippocampal gyrus (PHG) and then into the temporal pole (TP).

Deeper and more medial to the vILF, the lingula-amygdaloid (Li-Am) bundle was exposed. From the lingual cortex these fibres describe an arch-shaped bundle that follow the other ventral pathway until the temporal horn, where it turns around the tip of the temporal horn reaching the basolateral portion of the amygdaloid region (Figure 3(b)) [29]. Deeper with respect to the Li-Am, the optic radiation can be completely exposed on its ventral view until the primary visual cortex (V1) (Figure 3(c)).

### 3.3. Dissection of the Medial Surface

**Figure 4:**
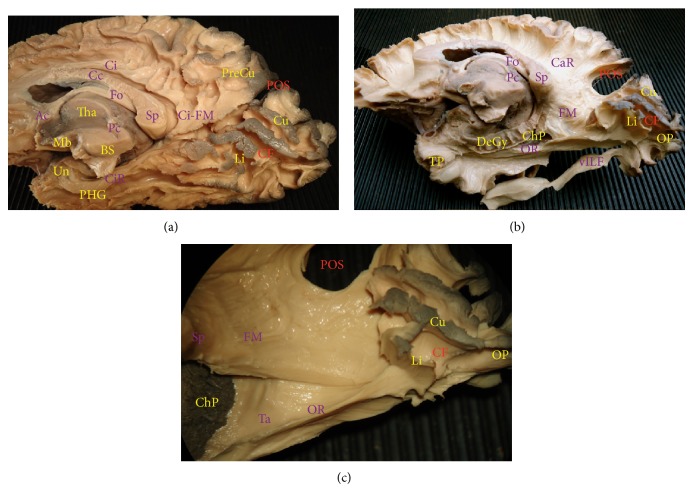
(a) Medial view of a right hemisphere after the initial dissection of cortex of the cingulate gyrus. The whole cingulum (Ci) is exposed with its extension from the subcallosal area to the parahippocampal gyrus (PHG) as the radiation of cingulum (CiR) until the region of the uncus. Posterior with respect to the splenium (Sp) of the corpus callosum (CC), a posterior component of the Ci reaches the cuneal and precuneal cortices, forming the most medial part of the forceps major (FM). (b) After the removal of the Ci and further dissection of the CC, the radiating callosal fibres (CaR) can be demonstrated. Once the hippocampus and the PHG have been removed, the lateral ventricle is opened with choroid plexus (ChP) shown at the medial portion of the trigone. The medial wall of the trigone is composed of callosal fibres of the FM, which end close to the optic radiation (OR) fibres on both sides of the calcarine fissure (CF). (c) The medial wall of the ventricle is opened cutting the FM fibres in order to expose the tapetal callosal fibres, which run within the lateral wall of the ventricle composing the deepest layer of the sagittal stratum of Sachs (SSS). The tapetal fibres end following the OR fibres on the medial surface of the cuneal and lingual regions, partially overlapped with the dorsal component of OR.* Fo: fornix; Ac: anterior commissure; Pc: posterior commissure; Mb: mammillary body; BS: brain stem; Tha: thalamus; TP: temporal pole; Un: uncus; DeGy: dentate gyrus; POS: parieto-occipital sulcus; CF: calcarine fissure; PreCu: precuneus; Cu: cuneus; Li: lingula; OP: occipital pole*.

Removing the cortex, subcortex, and short fibres of the cingulate gyrus exposes a group of fibres projecting in a longitudinal direction parallel and above the corpus callosum that forms the cingulum (Ci). At the level of the isthmus, where the commissural fibres of the forceps major cross in front of the cingulum fibres a horizontal component of the cingulum runs straight to cuneal-precuneal medial region (Figure 4(a)). Below this level, the cingulum courses near the most anterior part of the optic radiations and covers the inferior lip of the anterior part of the calcarine sulcus. Removal of the cingulum exposes the corpus callosum from the genu and rostrum anteriorly to the splenium posteriorly. At the level of the splenium, the fibres take a posterior oblique direction, forming the forceps major, which interconnects the parieto-occipital and calcarine regions (Figure 4(b)). The more lateral component of the callosal fibres represents the lateral wall of the trigone and atrium, reaching in a curvy shape the medial occipital surface cranial and anterior with respect to the optic radiation course (Figure 4(c)).

## 4. Discussion

The clinical evidences of variable epileptic propagation in OLE have been demonstrated in several studies with neurophysiological [36–39] and metabolic data (positron emission tomography (PET)) [9, 17, 40]. According to some recent series on surgical treatment of OLE, the position within the occipital lobe of an epileptic onset or the presence of a discrete lesion results in a specific pattern and semiology [9–18, 20, 23, 24, 41].

Even if visual aura is one of the hallmarks of OLE, described in up to 69% of the cases in some series [9, 23, 24, 26, 41–43], there is a significant spatial correlation with lateral and basal lesions with respect to the medial ones. According to the literature, elementary visual symptoms either positive (flashes and phosphenes) or negative (scotoma, hemianopsia, and amaurosis) are usually considered due to the stimulation or inhibition of the primary visual cortex and optic radiation [20, 44, 45]. The more the surrounding cortices are involved in the epileptic pattern, the visual symptoms usually become more complex with hallucinations, illusions, and delusions [8, 46, 47].

If we try to interpret the semiology of the OL seizures through the common reported patterns types we may argue that partial seizures with only occipital symptoms (such as seeing flashing lights or presented amaurosis) are basically restricted to the hyperactivation of the occipital intralobar connectivity. Dialeptic seizures, altered awareness associated to manual and/or alimentary automatisms, with or without epigastric aura reflect the temporal spread of the epileptic propagation. Early motor semiology such as tonic activity or hyperactive automatisms, secondary generalized tonic-clonic convulsions or rapid eye blinking or eyelid fluttering, and/or sensation of eye movement without any other movement are often reported as manifestation of seizure with frontal lobe involvement [8, 9, 23, 24, 41].

### 4.1. The Classical Pathways Revised

In the past, the different direction of the occipital epileptic patterns was classically related to the spatial relationship with the calcarine sulcus. For instance, medial occipital electric activity arising above the calcarine fissure usually propagates to the frontal lobe, while medial occipital electric activity arising below the calcarine fissure can propagate to the mesial temporal lobe [11, 19, 20]. Lateral occipital seizures would spread, according to this theory, to the parietal and lateral temporal lobes [13].

The functional connectivity of the occipital lobe is supported by projection pathways, which work in a serial activation and associative and commissural bundles that work in parallel networks.

The optic radiation is the prominent projection pathway in the visual system, conveying information from the lateral geniculate nucleus (LGN) with a specific direction towards the occipital region. Recent insights in tractographic segmentation of the optic radiation confirm in humans the existence of straight connection from LGN not only to V1 but also to the dorsal portion of the extrastriate visual cortices V2 and V3 [35]. This organization could explain the important number of elementary visual hallucinations (EVHs) like flashing lights or amaurosis due to the involvement of optic radiation even in just the dorsal portion of V2 and V3 [17, 20, 23]. Even if more detailed in the hierarchical segregation of the optic radiation fibres, however the emphasized position of V1 can only partially confirm the classical description of the other OLE patterns.

**Figure 5:**
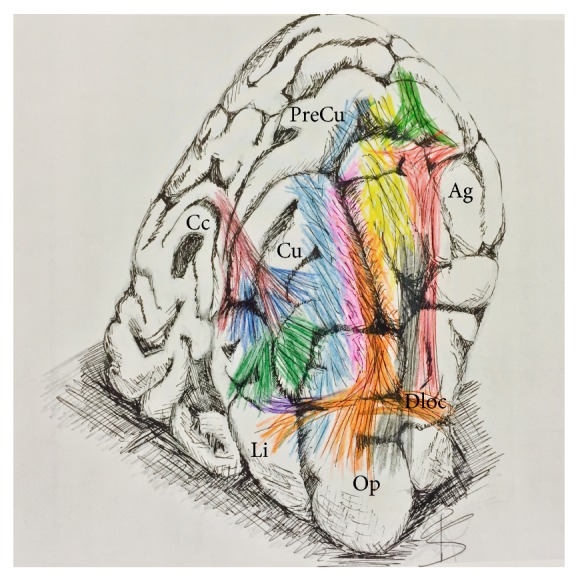
Artistic illustration of the white matter connectivity underlying the occipital region. The illustration shows a schematic reconstruction from a posterior coronal view the occipital region with all the major white matter bundles dissected and shown with different colours through the cortical layer. This illustration serves to summarize the complex subcortical organization, which can be significantly correlated to the semiology of the seizures involving each sublobar occipital region. A lesion on the lateral surface of the occipital lobe or at the temporo-occipital/parieto-occipital junction would probably spread to the parietal and frontal regions. The vertical component of the superior longitudinal fasciculus (vSLF/red), arcuate fasciculus or the horizontal superior longitudinal fasciculus (AF/yellow, hSLF/light green), and the vertical occipital fasciculus (VO/gray) can support this direction of propagation. Deep lesions may involve different pathways within the sagittal stratum of Sachs (SSS) such as inferior longitudinal fasciculus (ILF/orange), middle longitudinal fasciculus (MLF/pink), inferior fronto-occipital fasciculus (IFOF/light blue), or even the optic radiation (OR/dark green). According to the hierarchical segregation of the fibres, the epileptic propagation would spread to temporo-lateral areas, temporo-mesial areas, and/or frontal lobe. Moreover, a possible epileptic lesion in a dorsal-mesial location with respect to the OR and calcarine fissure would involve fibres of the cingulum (Ci/dark red) and callosal fibres of the forceps major-tapetum (Ta/dark blue) with a resultant ipsilateral and/or contralateral epileptic propagation. Finally, ventral-mesial deep lesions located below the calcarine fissure can involve the ventral portion of the IFOF (light blue) with a fast propagation to the frontal lobe or can recruit the ventral portion of the ILF (orange) and Li-Am bundle (LiAm/purple) with a fast temporo-mesial involvement (see the text for the functional considerations).* Ag: angular gyrus; Cc: corpus callosum; Cu: cuneus; Li: lingula; PreCu: precuneus; Op: occipital pole*.

From an anatomical point of view the inferior longitudinal fasciculus and the inferior fronto-occipital fasciculus have been classically considered the two major associative pathways responsible for the epileptic propagation in OLE to temporal and frontal areas, probably because of their close relationship with the optic radiation within the sagittal stratum of Sachs (Figure 5). However, from a functional point of view, several other white matter bundles may be responsible for the variable semiology of OLE.

Our dissection confirmed that the white matter connectivity of the occipital region is based on a very complex architecture with several longitudinal and vertical bundles closely interconnected on the mesial, lateral, and basal surface with a hierarchical segregation that may support the different directions of the epileptic propagation.

### 4.2. Dorsal Epileptic Pathways

According to the classical theory of Williamson and Bancaud [11, 19] an epileptic onset above the calcarine fissure from medial extrastriatal cortices (cuneal) would preferentially spread to frontal regions. A reasonable propagated onset through the IFOF terminations to the supplementary motor area (SMA), dorsolateral prefrontal cortex (DLPFC), and orbitofrontal cortex (OFC) would lead to clonic and/or tonic contraversion of the eyes and head or eyes only, forced closure of the eyelids, and palpebral jerks. However the dissection of the cuneal region showed other potential pathways that can be recruited by similar lesions on both medial and lateral portion. On the medial surface the fibres of the cingulum at the level of the cuneal-precuneal region can lead the epileptic propagation both to both frontal and temporo-mesial regions. The hyperactivation of this pathway with a frontal direction may explain the rapid secondary generalization to tonic-clonic seizures, while the temporo-mesial spread would support the role of this bundle in memory retrieving (Jamais vu and deja vu) described in patients with mesial-cuneal occipital lesions [20, 23, 41]. Between the cingulum and the optic radiation the posterior commissural and the callosal fibres of the forceps major represent a fast interhemispheric pathway able to propagate the electric signal to the contralateral side with a subsequent possible secondary recruitment of temporal and/or frontal regions. The prolonged and abnormal activation of these pathways may be responsible for the appearance of the so-called “mirror focus” on the contralateral hemisphere in intractable epilepsy [48].

Lateral to the optic radiation and the inferior fronto-occipital fasciculus occipital terminations, within the SSS, the middle longitudinal fasciculus (MLF) can be responsible for the fast epileptic spread to lateral temporal region. Reported symptoms like tinnitus, vertigo, and vertiginous visual sensations can be due to the epileptic propagation to the posterior portion of superior temporal gyrus through the MLF fibres. More lateral and inferior with respect to the middle longitudinal fasciculus, cuneal terminations of the dILF can support the onset with the visual aura reported in few medial lesions [9, 20, 23, 24]. The dILF with its double origin (cuneal and dorsolateral occipital cortex) may facilitate the convergence of the spreading from both lateral and medial areas to the occipito-temporal lateral areas and temporal pole. Simple illusions may be supported by this bundle within the temporo-occipital region. Objects can be described as distorted and eventually changed in size (macropsia or micropsia), shape (metamorphopsia), illumination, colour, or clarity. Lines may appear wavy (dysmorphopsia), objects may appear inclined (plagiopsia), and there may be a loss of colour (achromatopsia) [8, 46, 47].

### 4.3. Lateral Epileptic Pathways

Lateral occipital epileptic onset with respect to the V1 was classically associated to parietal propagations [11, 19, 20]. According to the white matter connectivity of the parieto-occipital (PO) region, we may argue that this classical pattern is at least incomplete for two reasons.

First, a parietal recruitment can easily lead to a frontal propagation with early sensory-motor semiology. Frontal involvement can indeed occur in a parallel manner for both medial and lateral occipital lesions [9, 20, 23, 24, 41]. Particularly in cases of associated numbness to the contralateral hemibody [20], a perisylvian propagation can be hypothesized trough the AF/SLF complex, which connects the extrastriate (V4-V5) lateral cortices through the parietal region (angular gyrus (AG)) and then to premotor and motor cortical areas. In particular, this pathways end in the frontal eye fields (FEF), the supplementary eye fields (SEF), and the dorsolateral prefrontal cortex (DLPC). The facilitated propagation through this so-called dorsal visual stream, the “Where” stream [49], may be responsible for complex visual hallucinations and illusions or postictal negative symptoms such as the inability to revisualize objects or scenes (disorders of mental imagery), strange or unreal visual experiences (de-realization), out-of-body experiences, or motion hallucinations [46].

The second reason is that not only a lateral occipital onset can preferentially spread to parietal and then frontal areas but also temporo-occipital basal lesions can show a parietal recruitment during the epileptic propagation. On this note the vertical occipital fasciculus can represent a parallel hyperactivated pathway supporting the parietal propagation from more basal onset involving the fusiform gyrus. This additional connection between basal and lateral/parietal region could represent an anatomical-functional bridge between the “What” ventral pathway and the “Where-How” dorsal pathways for vision [49]. Hence, complex hallucinations and illusions both involving words and images could be supported by this white matter pathway [50].

### 4.4. Ventral Epileptic Pathways

Based on the surgical results for lesions involving the infero-lateral surface, the specific epileptic propagation to the anterior and lateral temporal regions could be supported by the segregated longitudinal WM architecture of this area [9, 20, 23, 24, 41]. Not in all the cases the mesial areas are involved by the epileptic propagation differently from classical theory regarding the relationship with the calcarine fissure [11, 19]. The analysis of seizures semiology in this area so rich in specialized cortical regions (lateral infero-temporal multimodal area (LIMA) [51]; visual word form area (VWFA) [52, 53]; cortices activated by several visual inputs such as face (fusiform face area (FFA)), object (visual object form area (VOFA)), houses, animals (lateral occipital, LO, and V3–V7) [54]) can give us important information about the intrinsic white matter connections involved in the temporal propagation pattern. For instance, face hallucinations and illusions characterized by distorted facial features (prosopometamorphopsia) are likely to relate to a region specialized for face features on the lateral convexity of the occipital lobe (occipital face area, subserved by the dILF) while hallucinations of normal faces or facial intermetamorphosis (a change in the visually perceived identity of a face) are likely to relate to activity within an area specialized for faces on the ventral occipito-temporal surface (fusiform face area, subserved by the vILF) [55]. In our opinion, simple illusions like macropsia or micropsia, metamorphopsia, illumination, colour or clarity, dysmorphopsia, plagiopsia, and achromatopsia may be explained by the loss of balance of this longitudinal temporo-occipital network. On the other hand a propagation from the basal region to the lateral occipital and parietal region (through the VO, vSLF, and AF) would create complex illusions like objects that appear disorientated in distance (macroproxiopia, microtelepsia), or be distant and minute (teleopsia), or that have a loss or enhancement of stereoscopic vision or even persistent or recurrent (palinopsia) (see [6–8] for reviews).

However, if the seizure spreads to the mesial temporal lobe such as the parahippocampal gyrus (PHG), automatisms and impaired awareness may occur [8, 38] and according to the white matter architecture of this area the vILF may be responsible for this semiology. More medial and deeper to the vILF, the Li-Am bundle represents a direct connection between the lingual gyrus and the basolateral portion of the amygdaloid region. Confirming the already reported data about the fast activation of amygdaloid region and extrastriatal visual cortices [36, 37] this fast and short connection may explain why many patients [20, 56] with lesions involving the lingual gyrus experience an ictal semiology of fear and anxiety.

To summarize, this paper supports the description of a very complex white matter architecture underneath the occipital region. Considering each possible direction of electric propagation, we describe different anatomical pathways, which may support in parallel the spread from extrastriatal cortices to frontal, parietal, or temporal regions. All this connectivity is supported by white matter bundles that are closely interconnected with partially overlapping terminations. Moreover, each pathway except the OR represents a bidirectional network and for this reason able to repropagate the epileptic signal back to the other extrastriate cortices. On this note, according to the incredible number of patients with visual aura and to the absence of this phenomenon within the primary visual cortex, we may claim that visual aura can be facilitated by the hyperactivation of the associative pathways once the epileptic propagation is directed back to the occipital region and/or enhanced by the short intralobar fiber systems. This mechanism may be valuable also in explaining the visual symptoms (either simple or complex) reported in patients with a confirmed extraoccipital epileptic focus (frontal, temporal, or parietal) [26, 57–61].

Fort this reason even if complex, the possibility to predict or specifically localize the way of epileptic propagation should always be based on the match between the semiology and the underlying white matter pathways.

Further studies should merge clinical, neurophysiological, neuroradiological, and anatomical data in order to define the best therapeutic options, minimizing complications in patients with OLE.

## 5. Conclusion

New insights into the occipital white matter architecture support a more complex functional connectivity based on both vertical and longitudinal bundles which are specialized, segregated, and interconnected and are able to propagate the electric signal bidirectionally. A more comprehensive understanding of the occipital white matter connectivity merged with neuroradiological, functional, and neurophysiological studies may better clarify the incredible variability of symptoms related to an epileptic onset in the occipital region.
